# Bio-Priming of Soybean with *Bradyrhizobium japonicum* and *Bacillus megaterium*: Strategy to Improve Seed Germination and the Initial Seedling Growth

**DOI:** 10.3390/plants11151927

**Published:** 2022-07-25

**Authors:** Dragana Miljaković, Jelena Marinković, Gordana Tamindžić, Vuk Đorđević, Branislava Tintor, Dragana Milošević, Maja Ignjatov, Zorica Nikolić

**Affiliations:** 1 Department of Microbiological Preparations, Institute of Field and Vegetable Crops, 21000 Novi Sad, Serbia; jelena.marinkovic@ifvcns.ns.ac.rs (J.M.); branislava.tintor@ifvcns.ns.ac.rs (B.T.); 2 Laboratory for Seed Testing, Institute of Field and Vegetable Crops, 21000 Novi Sad, Serbia; gordana.tamindzic@ifvcns.ns.ac.rs (G.T.); dragana.milosevic@ifvcns.ns.ac.rs (D.M.); zorica.nikolic@ifvcns.ns.ac.rs (Z.N.); 3 Department of Legumes, Institute of Field and Vegetable Crops, 21000 Novi Sad, Serbia; vuk.djordjevic@ifvcns.ns.ac.rs; 4 Department of Vegetable and Alternative Crops, Institute of Field and Vegetable Crops, 21000 Novi Sad, Serbia; maja.ignjatov@ifvcns.ns.ac.rs

**Keywords:** bio-priming, seed and seedling quality, soybean, stress, PGPR

## Abstract

Bio-priming is a new technique of seed treatment that improves seed germination, vigor, crop growth and yield. The objective of this study was to evaluate the effectiveness of *Bradyrhizobium japonicum* (commercial strains) and *Bacillus megaterium* (newly isolated strains) as a single inoculant and co-inoculant during seed bio-priming to improve seed germination and initial seedling growth of two soybean cultivars. The treated seeds were subjected to germination test (GT), cold test (CT) and accelerated aging test (AAT). *B. megaterium* significantly improved all parameters in GT and CT; final germination, shoot length, root length, root dry weight, and seedling vigor index in AAT, as compared to control. In addition, co-inoculation significantly increased all parameters except shoot dry weight in GT; all parameters in CT; germination energy, shoot length, root length, and seedling vigor index in AAT, in comparison to the control. Moreover, *Br. japonicum* significantly improved the germination energy, shoot length, shoot dry weight, root dry weight, and seedling vigor index in GT; all parameters in CT; shoot length, root length, and seedling vigor index in AAT, compared with non-primed seeds. Thus, *B. megaterium* strains could be used in soybean bio-priming as a potential single inoculant and co-inoculant, following proper field evaluation.

## 1. Introduction

Soybean (*Glycine max* (L.) Merrill) is one of the most important leguminous crops, with the highest protein content—around 40%, and the second highest oil content—around 20% [1]. Statistical data showed that the world’s soybean production increased approximately 12-fold from 1961 to 2019. Soybean is grown at an overall surface of 122 million ha, with total annual production of 339 MMT [2]. In recent decades, crop scientists and farmers have made concerted efforts to reach the desired soybean growth and productivity, which has led to the production of plants with desirable traits, such as increased grain yield, improved seed quality, and enhanced adaptation to environmental changes [3].

Seeds are the most important determinants of efficient plant growth and development, fundamental to crop production. Successful germination and seedling establishment is crucial to achieving high biomass and high yield. The desired germination and seedling development can be inhibited by untimely sowing and low seed quality, accompanied by adverse post-sowing conditions [4]. An important ability of seeds is to germinate and establish seedlings rapidly and uniformly (seed vigor), especially under conditions which could challenge germination [5,6]. Soybean seed germination potential is often impaired prior to sowing. Problems in soybean seed quality occur during pre- and postharvest periods, and mostly include seed deterioration due to adverse environmental conditions, and seed diseases due to numerous pathogens [7]. In order to improve soybean seed quality, especially under stressful conditions and/or diseases, numerous researchers have explored different strategies, including pre-sowing seed treatments with beneficial microorganisms [8]. The development of microbial inoculants using plant growth-promoting rhizobacteria (PGPR) is increasingly regarded as an environmentally-friendly alternative to the extensive use of synthetic chemicals in agriculture [9]. The use of PGPR is of particular importance in organic production, where the use of synthetic fertilizers and pesticides must be avoided in order to improve the quality of agricultural soils and products. Furthermore, using good quality seeds is an additional production imperative due to limited seed availability and other issues regarding organic production. Bio-priming is a seed priming method, which enables bacterial adherence and adaptation to the seeds, improves colonization of rhizosphere and plant tolerance to various biotic and abiotic stresses, such as seed and soilborne pathogens and adverse environmental conditions [10]. PGPR application through bio-priming involves soaking the seeds in liquid bacterial culture suspension for a particular period, initiates physiological processes inside the seed and enhances the bacterial abundance in the spermosphere, while preventing plumule and radicle emergence until the seed is sown [10,11].

A large number of PGPR have been reported to stimulate plant growth by producing plant hormones and increasing the availability of nutrients in the soil [12]. Bacteria characterized as PGPR also contribute to the suppression of plant pathogens through competition, antibiosis, parasitism or predation and induction of systemic resistance (ISR) [13]. Furthermore, PGPR can help maintain plant growth and development under various stress conditions that limit crop productivity [14]. Beneficial effects of PGPR on germination, growth and yield have been reported in many field and vegetable crops, including soybean [15]. *Bradyrhizobium* species, including *Bradyrhizobium japonicum*, represent the most common symbiotic bacteria forming nitrogen-fixing nodules on soybean [16]. Due to this symbiotic association, the amounts of N_2_ fixed by soybean range from zero to 337 kg N ha^−1^ of arable soil annually, depending on rhizobial activity [17]. In addition to its high effectiveness in nitrogen fixation, bradyrhizobia share a multitude of other properties with PGPR. *Bacillus* species, including *Bacillus megaterium*, are the most abundant among soil and plant-associated bacteria [18]. Bacilli usually display several actions of PGPR, including plant growth-promoting properties as well as antagonism against plant pathogens [19]. Moreover, *Bacillus* spp. form spores that are able to resist very harsh environments [20]. Synergistic actions of these bacteria could be highly advantageous in selecting the best possible inoculant for soybean. Considering the benefits to plant growth associated with both symbiotic bacteria and PGPR, co-inoculation of soybean might improve seed quality, especially under suboptimal conditions, like those which may occur in the field and/or storage. Nonetheless, there is a limited number of commercial products and a lack of information about the use of *B. megaterium* as seed bio-priming treatment, singly or in combination, in soybean production. Therefore, the aim of this study was to examine the impact of single inoculation and co-inoculation with *Br. japonicum* and *B. megaterium* during seed bio-priming on seed germination and initial seedling growth of two soybean cultivars under optimal and stressful conditions.

## 2. Results and Discussion

*B. megaterium* strains used in this study were initially isolated from soil samples collected from different sites of the Vojvodina Province (northern Serbia) and identified based on 16S rDNA sequencing (Table 1).

### 2.1. Plant Growth-Promoting Characterization of Bacillus Megaterium Strains

Multiple plant growth-promoting (PGP) properties of the *B. megaterium* strains are presented in Table 2. All *Bacillus* strains were able to produce indole-3-acetic acid (IAA) in medium with L-tryptophan (8.00–15.65 µg mL^−1^). Among the seven *B. megaterium* strains, five were able to solubilize phosphorus, seven were positive for phosphate mineralization and able to grow on N-free medium, and six strains were capable of producing siderophores. Similarly, Haque et al. [21] reported that all rhizobacterial strains including *B. megaterium* expressed production of IAA, solubilization of nutrients (P, K, Zn), production of siderophores, and other PGP activities. Moreover, Nascimento et al. [22] found genes in the genome of *B. megaterium* that participate in the biosynthesis of auxins and cytokinins, stress resistance, antagonistic activities, and other PGP traits.

Although the newly-isolated bacterial strains tested in this study generally had good PGP abilities, four strains of *B. megaterium*: B8, B12, B15 and B17 were selected due to the highest potential for plant growth promotion. Dual culture assay demonstrated that the chosen *B. megaterium* strains were compatible with each other. Furthermore, compatibility assay did not show any antagonistic actions among *Br. japonicum* and *B. megaterium* strains.

### 2.2. Effect of Seed Bio-Priming Treatments on Seed Germination and Initial Seedling Growth of Soybean

Seed germination, together with seed vigor, represents a key factor in determining crop yield [23]. Seed quality also affects the rate and uniformity of emergence as well as the rate of initial plant growth [24]. Numerous factors, such as conditions of development and storage, affect seed germination and seed vigor [25]. Three laboratory tests—germination test, cold test, and accelerated aging test—were performed in order to better characterize seed quality in relation to the applied seed bio-priming treatments. Germination test determines the percentage of viable seeds and their ability to produce normal seedlings under optimal temperature, moisture and light conditions. The cold test estimates seed quality performance under unfavorable conditions such as low temperature, while the accelerated aging test assesses the ability of a seed lot, as a specified quantity of agricultural seeds, from the same harvest and of the same origin and quality, to perform under stress conditions, such as high temperature and high relative humidity. The germination test can be used to predict field emergence under optimal conditions, whereas the results of cold and accelerated tests are often better correlated with field emergence under adverse environmental conditions [26]. The effect of cultivar, treatment and their interaction on seed germination and initial seedling growth is presented in Table 3 and Supplementary Materials Table S1. Soybean parameters were significantly influenced by seed bio-priming treatments, both in optimal and stressful conditions (Table 3a–c). The cultivar had a significant effect on shoot length, root length, root dry weight, and seedling vigor index in optimal conditions (Table 3a); shoot length, root length, shoot dry weight, root dry weight, and seedling vigor index in cold stress (Table 3b); final germination, abnormal seedlings, shoot length, root dry weight, and seedling vigor index in double stress (Table 3c). Furthermore, cultivar × treatment interaction significantly altered all parameters of normal seeds (Table 3a); abnormal seedlings, shoot length, root length, shoot dry weight, and seedling vigor index of cold-treated seeds (Table 3b); germination energy, final germination, abnormal seedlings, shoot length, root length, root dry weight, and seedling vigor index of aged seeds (Table 3c).

The effect of seed bio-priming treatments on seed germination and initial seedling growth of the soybean cultivars in the germination test is presented in Table 4a. On average, all bacterial treatments positively affected the examined soybean parameters compared to the control. A significant effect of all treatments in relation to the control was observed in germination energy, shoot length, root dry weight, and seedling vigor index. Furthermore, significant differences between *B. megaterium* or co-inoculation and the control were noted in final germination, abnormal seedlings, and root length, while single treatments had a significantly higher effect compared to control on shoot dry weight. *Bacillus* treatment led to the highest increase in final germination (3.5%), root length (8.9%), shoot dry weight (18.9%), and seedling vigor index (12.5%) compared to the control. The highest increase in germination energy (3.4%), and shoot length (8.2%) in comparison to the control was recorded with *Br. japonicum*. However, *Br. japonicum* did not improve final germination and abnormal seedlings. The highest decrease in abnormal seedlings (−2.8%), and increase in root dry weight (70.2%) related to the control was observed after co-inoculation. Nevertheless, the mentioned treatments, that showed the highest and significant effect compared to the control, did not differ significantly in relation to certain individual treatments. Only *Bradyrhizobium* had significantly lower effect compared to *Bacillus* and co-inoculation on root length and seedling vigor index, and single treatments had significantly higher effect compared to co-inoculation on shoot dry weight (Table 4a).

The analysis of average soybean parameters across cultivars revealed significant and positive effects of all bacterial treatments in relation to their respective controls, in the cold-treated seeds (Table 4b). However, there was not significant difference between treatments, except in abnormal seedlings where *Br. japonicum* treatment differed from other treatments. The highest increase in final germination (6%) and decrease in abnormal seedlings (−4.8%) in comparison to the control was obtained from inoculation with *Br. japonicum*, while the highest increase in root length (43.7%) and root dry weight (49.1%) was observed after inoculation with *B. megaterium*. A combined treatment with *Br. japonicum* and *B. megaterium* led to the highest improvement in shoot length (35.1%), shoot dry weight (9.3%), and seedling vigor index (48.5%) compared to the control (Table 4b).

On the other hand, the cultivar average revealed a positive and significant effect of single and co-inoculation treatments, compared to the control, on shoot length, root length, and seedling vigor index under double stress (Table 4c). However, consistent improvements in other seedling parameters of aged seeds were not found. Moreover, *Bacillus* treatment had significant influence, in comparison to the control, on final germination and root dry weight, while co-inoculation significantly altered germination energy. The highest increase, compared to the control, in final germination (3.3%), root length (43.8%), root dry weight (17.2%), and seedling vigor index (32.4%) was obtained from inoculation with *B. megaterium*. Co-inoculation had the highest impact on germination energy (3.1%), and shoot length (19.8%) in comparison to the control (Table 4c). Bacterial treatments did not increase shoot dry weight. Furthermore, *Br. japonicum* and co-inoculation did not improve germination, while all treatments increased abnormal seedlings (Table 4c).

The optimal temperature for soybean germination is 25 °C (above 10 °C). Stressful conditions, such as high or low temperatures and high humidity, can decrease germination and extend germination time, primarily due to cell membrane damage and electrolyte leakage. Low temperatures, especially during the first few days of the germination period, have a major effect on seed germination and initial growth, as shown later in soybean development and yield. According to Szczerba et al. [27], low temperature inhibited germination and caused a decrease in germination vigor, fresh and dry weight of aboveground parts, and yield parameters of four soybean cultivars. Moreover, high temperature and high humidity decreased seed quality of two soybean cultivars [28]. In the present study, abnormalities observed in soybean seedlings, such as stunted, retarded and deeply broken primary root, were probably due to physiological and biochemical causes and may have been associated to adverse conditions. Low temperatures during early germination can reduce the imbibition rate, mitochondrial respiration, and embryo expansion in soybean [29]. On the other hand, high temperature and high humidity can lead to seed deterioration, such as seed abnormalities, reduction in germination, seed vigor and quality [30]. A reduction in the percentage of abnormal seedlings under low temperature observed in this study can be attributed to the effect of seed bio-priming treatments. It was found that PGPR regulate morpho-physiological, biochemical and molecular responses in plants to combat the adverse effects of various environmental stresses [31]. These bacteria were able to produce plant hormones and modulate the hormonal balance and plant response to stress. However, the increase in abnormal seedlings of inoculated seeds under high temperature and high humidity may be connected with the moisture content after bio-priming, along with the conditions of performing this test. The hydrophilic nature of high protein content and high oil content contribute to more absorption of water and the deterioration of soybean seeds, while a high temperature accelerates the rate of biochemical processes, which cause more rapid deterioration in seeds with high moisture content [7]. The seed membrane system and breathing capacity is damaged, which in turn leads to lower energy output and a decrease in enzymatic activity. All these changes result in lower germination velocity, inhibited seedling growth and development, decreased tolerance to adverse environmental conditions, and higher proportion of abnormal seedlings [32].

**Figure 1:**
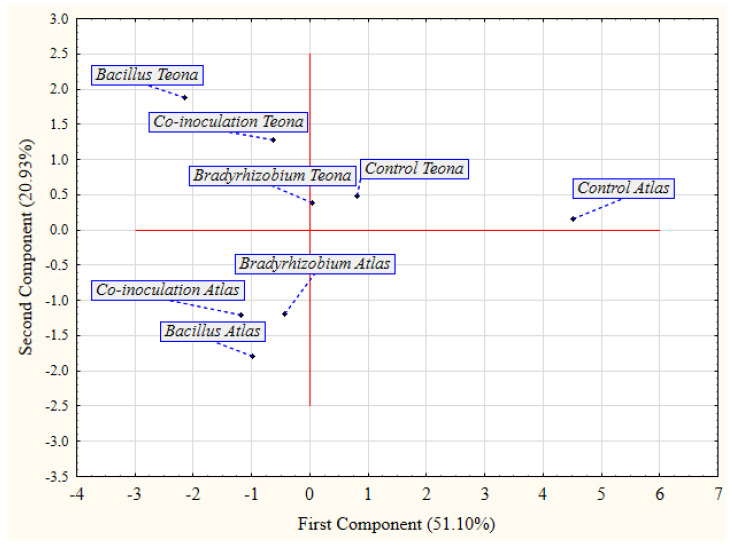
Principal component analysis (PCA) for the effect of seed bio-priming treatments on the examined parameters of soybean cultivars in germination test.

**Figure 2:**
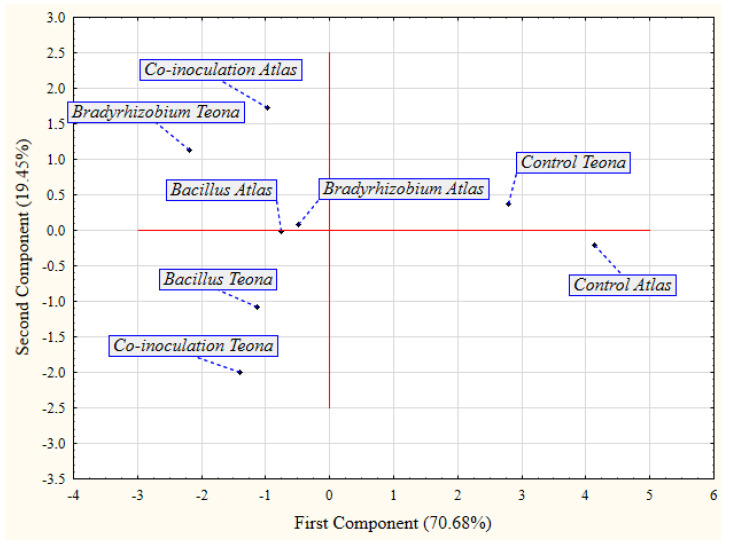
Principal component analysis (PCA) for the effect of seed bio-priming treatments on the examined parameters of soybean cultivars in cold test.

**Figure 3:**
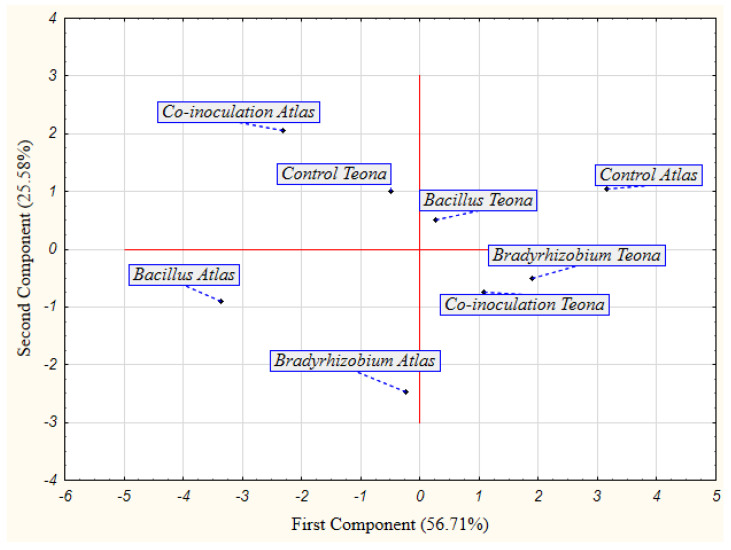
Principal component analysis (PCA) for the effect of seed bio-priming treatments on the examined parameters of soybean cultivars in accelerated aging test.

Furthermore, the association between bacterial treatments and cultivars in optimal and adverse conditions is illustrated by the biplot of the principal component analysis (PCA) (Figure 1, Figure 2 and Figure 3). In optimal conditions (Figure 1), bacterial treatments were clearly separated from control treatments, except *Bradyrhizobium* treatment in cv. Teona, due to its lower effect on the examined soybean parameters. Additionally, PCA showed that cultivars were separated from each other within the same group. Hence, it was confirmed that *Bacillus* and co-inoculation treatments had a better effect than *Bradyrhizobium* on both cultivars.

According to the PCA for bacterial treatments and cultivars under cold stress (Figure 2), bacterial treatments were classified into the same group, while control treatments were clearly separated in the second group. Co-inoculation was disjointed from single inoculation treatments in cv. Atlas, due to its better effect on the examined parameters. Additionally, *Bradyrhizobium* was clearly separated from *Bacillus* and co-inoculation, being the optimal treatment in cv. Teona.

Based on the PCA for bacterial treatments and cultivars under double stress (Figure 3), bacterial treatments in cv. Atlas were clearly separated from bacterial treatments in cv. Teona, which indicates a different response of cultivars to bacterial treatments. Furthermore, bacterial treatments of each cultivar were clearly separated from their control treatments, which confirms a predominantly positive effect of bacterial treatments in cv. Atlas, and a contrary effect in cv. Teona. The PCA results (Figure 1, Figure 2 and Figure 3) are in line with the results presented in Supplementary Materials Tables S2–S4.

The results emphasized the role of inoculants in establishing soybean tolerance to stressful conditions such as low or high temperature and high humidity. However, the different response of cultivars to inoculation (Supplementary Materials Tables S2–S4), especially noticeable in adverse conditions, implies the possibility of improving seed germination and seedling growth of soybean by selecting the appropriate seed bio-priming treatment for each cultivar in field testing using several seed lots of each cultivar, preferably all high germinating but with different vigor levels, and according to specific environmental conditions. Generally, PGPR activity is largely determined by fluctuations in environmental factors [33]. *B. megaterium* strains have the ability to grow at a temperature range 3–45 °C [34], whereas *Br. japonicum* strains tolerate 15–20 °C and below well, although less frequently [35]. However, bacterial growth and activity were obviously not influenced by suboptimal conditions in the current study. A higher effect of *B. megaterium* as a single and/or co-inoculant compared to the sole application of *Br. japonicum*, especially under high temperature and humidity conditions, can be attributed to their ability to produce spores, grow and remain active under stress. Concurrently, survival and efficacy of bacterial strains, particularly on aged seeds, indicates the possibility for prolonged storage of bio-primed seeds.

Additionally, correlation analysis confirmed positive effects of seed bio-priming treatments under both optimal and unfavorable conditions, with the largest impact under cold stress. Overall, a positive interrelationship was established between germination and other parameters, with an exception of abnormal seedlings (Table 5). In optimal conditions, final germination was significantly related with shoot dry weight, root dry weight, and seedling vigor index (Table 5a). Furthermore, final germination was significantly correlated with shoot length, root length, root dry weight, and seedling vigor index of the cold-treated seeds (Table 5b). Moreover, a significant dependence was observed between germination parameters, shoot length, and the seedling vigor index of aged seeds (Table 5c). PGPR activities are pivotal in early developmental stages, such as germination and initial seedling growth. Thus, seed bio-priming with PGPR could lead to increased biomass and grain production in later developmental stages.

The current study, as well as several other studies, reported the positive effects of using PGPR as co-inoculants of soybean with *Br. japonicum*, in order to improve plant growth under adverse conditions. Namely, in a meta-analysis of studies from 1987 to 2018, Zeffa et al. [36] summarized that co-inoculation of soybean with *Bradyrhizobium* and PGPR (*Azospirillum*, *Bacillus*, *Pseudomonas*, *Serratia*) improves plant development, increases nodulation, and facilitates nutritional limitations and potential stresses during plant growth. Moreover, co-inoculation of soybean with *Br. japonicum* and *Bacillus* strains, which were shown to have PGP activity, increased nodule number, total biomass, total nitrogen, and yield, under the conditions of low root zone temperatures [37]. Cardarelli et al. [8] reported that the application of beneficial microorganisms to the seeds of the major crops resulted in increased germination, seedling vigor, biomass, and the ability to overcome stress both during and after emergence. Generally, seed bio-priming with PGPR improves seed quality, plant growth and stress tolerance through production of regulatory substances, nutrient enhancement, and plant protection [38]. It initiates physiological processes linked to increased activity of hydrolytic and detoxifying enzymes, ROS, modifications of hormone levels, and gene expression in plants [39]. The beneficial effects of PGPR are predominately based on their ability to produce auxins, cytokinins, gibberellins, salicylic acid and abscisic acid. PGPR keep the ability to regulate hormones and metabolism in plants, specifically in biochemical processes that can preclude the detrimental effects of abiotic and biotic stresses [40]. Diverse PGPR, including *Bacillus* strains tested in this study, produce IAA. It was found that PGPR use this hormone to interact with plants as part of their colonization strategy. Being the first contact between the bacteria and the seed, IAA penetrates the seed coat with water, promotes seed germination, enhances and regulates vegetative growth and development, affects biosynthesis of various metabolites, resistance to stressful conditions, photosynthesis, etc. [41]. As a result, bacterial IAA increases root length and surface area, and thereby improves the attainability of nutrients. Furthermore, previous studies point to the existence of presumable crosstalk among IAA and other phytohormones that mediate and improve tolerance responses in plants [42]. For instance, Khan et al. [43] found that IAA-producing *Bacillus* strains improved biomass and stress tolerance of soybean plants, while modulating abscisic acid (ABA) and salicylic acid (SA) levels. Additionally, PGPR have the ability to solubilize/mineralize phosphorus, and produce siderophores, which was also confirmed for *B. megaterium* strains used this study. This study confirmed that single inoculation and co-inoculation of soybean with *B. megaterium* improved seed germination and initial seedling growth, probably due to the production of IAA and other PGP activities. Similarly, *Bacillus velezensis* increased germination, root length and root surface of soybean compared to control, while strain genome analysis revealed the presence of genes linked to root colonization and PGP ability [44]. Single inoculation and co-inoculation of soybean with *Br. japonicum* and *Bacillus amyloliquefaciens* promoted early seedling growth and significantly improved nodulation, probably due to the production of high levels of auxin, gibberellins and salicylic acid by the *Bacillus* strains [45]. 

## 3. Materials and Methods

### 3.1. Bacterial Cultures

This study used *Br. japonicum* (BJ) and *B. megaterium* (BM) strains from the collection of the Laboratory for Microbiological Research of the Institute of Field and Vegetable Crops, Novi Sad (IFVCNS), Serbia. Strains of *Br. japonicum* are commercially used as microbiological fertilizer in the production of soybean in Serbia. Strains of *B. megaterium* were isolated in 2015–2016 from soil samples collected from different locations in northern Serbia (B8: Rimski Šančevi, 45°20′00″ N, 19°51′00″ E; B9: Perlez, 45°12′23″ N, 20°22′56″ E; B12: Rumenka, 45°17′21″ N, 19°44′23″ E; B14: Bački Petrovac, 45°21′36″ N, 19°35′26″ E; B15: Pančevo, 44°54′00″ N, 20°40′00″ E; B16: Futog, 45°14′17″ N, 19°42′22″ E; B17: Kovilj, 45°13′24″ N, 20°01′11″ E). Soil samples included the agricultural soil (rhizosphere and bulk soil), as well as non-agricultural soil, which differed in chemical and physical properties. Isolation was performed using serial dilution and streak plate methods on nutrient agar (NA). *Bacillus* strains were morphologically characterized and identified by 16S rDNA sequencing (Table 1). Morphological characterization of *Bacillus* strains included the examination of shape, size, color, opacity, homogeneity, and texture of colonies, as well as shape, size and mobility of cells. DNA from the 24-h-old bacterial cultures was extracted using a DNeasy Mini Kit (QIAGEN Inc., Hilden, Germany), while the amplification of 16S rDNA gene fragments of *Bacillus* strains was performed using primers 27F (AGAGTTTGATCMTGGCTCAG) and 1492R (TACGGYTACCTTGTTACGACTT) [46]. Amplifications were conducted on the Eppendorf Mastercycler PCR device (Eppendorf, Hamburg, Germany). Cycling parameters for PCR were 94 °C for 5 min, followed by 30 cycles of 94 °C for 30 s, 50 °C for 1 min, 72 °C for 30 s each, and then 72 °C for 7 min. Amplicons were determined using electrophoresis on 1.5% agarose gel containing ethidium bromide (0.5 µg/mL). Purification and sequencing of the PCR-amplified DNA fragments were conducted in the Macrogen Europe Laboratory, Amsterdam, Netherlands. All sequences were deposited in the NCBI (National Center for Biotechnology Information) [47]. *Bradyrhizobium* strains were cultured in yeast extract mannitol broth (YEMB) and incubated at 28 ± 2 °C and 120 rpm (Edmund Bühler SM-30 B, Bodelshausen, Germany) for 72 h. *Bacillus* strains were grown in nutrient broth (NB) and incubated at 30 ± 2 °C and 120 rpm for 24 h. Each strain of *Bradyrhizobium* and *Bacillus* was grown individually, and then mixed in equal proportions to form the mixtures for further examination. A culture suspension of each mixture was adjusted to have a final concentration of 10^9^ colony forming units per mL (CFUs/mL).

### 3.2. Screening of Bacillus megaterium Strains for Plant Growth-Promoting Properties

For quantitative analysis of indole-3-acetic acid (IAA) production, 1 mL of overnight grown culture of each *Bacillus* strain was inoculated in NB supplemented with 500 µg/mL of L-tryptophan (HiMedia, Mumbai, India) and incubated at 30 ± 2 °C for 24 h. The supernatant was mixed with Salkowski reagent (FeCl_3_-HClO_4_: 1 mL of 0.5 M ferric chloride solution in 49 mL of 35% perchloric acid) in a ratio of 1:2 (supernatant/reagent *v*/*v*). Development of a pink color after 20 min at room temperature indicated the production of IAA. Indole production was measured by spectrophotometric absorption (UV/VIS Cary 60 E, Agilent, CA, USA) at 530 nm [48]. The phosphate solubilization and phosphate mineralization were determined on inorganic phosphorus (Pi) culture medium and organic phosphorus (Po) culture medium [49] supplemented with tricalcium phosphate (HiMedia, Mumbai, India) and lecithin (Thermo Fisher Scientific, Waltham, MA, USA), respectively. The potential of isolates to grow without nitrogen was determined on Döbereiner culture medium [50]. Bacterial ability to produce siderophores was determined on chromeazurol S (HiMedia, Mumbai, India) medium by detecting changes of the color zones from green-blue to orange [51]. The compatibility of *Br. japonicum* vs. *B. megaterium*, and/or *B. megaterium* strains with each other was tested by dual culture assay on yeast extract mannitol agar (YMA) and/or NA plates. All determinations were performed in triplicate (Table 2).

### 3.3. Preparation of Soybean Seeds

Seeds of two cultivars of soybean (*Glycine max* L.), namely Atlas and Teona, developed at the Soybean Department, IFVCNS were used in the present study. Seeds were surface disinfected in 2% sodium hypochlorite (Sigma Aldrich, St. Louis, MO, USA), washed with sterile distilled water four times, and then dried back on sterile filter paper under aseptic conditions to their original weight. In addition, seeds were soaked in 150 mL of bacterial culture suspension per each test and treatment (4 × 100 = 400 seeds) for germination and accelerated aging tests, and 75 mL per treatment (4 × 50 = 200 seeds) for cold test. Three bacterial treatments were tested: *Br. japonicum* (BJ), *B. megaterium* (BM), and *Br. japonicum* + *B. megaterium* (BJ + BM), with the inoculum rate 2 × 10^9^ CFUs/g. The ratio of the strains in their mixtures in single inoculation treatments, as well as the ratio of the *Bradyrhizobium* and *Bacillus* mixtures in the co-inoculation treatment, was 1:1 (75 mL: 75 mL per 150 mL and 37.5 mL: 37.5 mL per 75 mL). *Bradyrhizobium* mixture contained 6 strains of *Br. japonicum*: the amount of each strain in single and co-inoculation treatments was 25 mL and 12.5 mL, respectively, per 150 mL (germination and accelerated aging tests), and half less milliliters per 75 mL (cold test). *Bacillus* mixture contained 4 strains of *B. megaterium*: the amount of each strain in single and co-inoculation treatments was 37.5 mL and 18.75 mL, respectively, per 150 mL (germination and accelerated aging tests), and half less milliliters per 75 mL (cold test). Non-primed seeds were used as the control. Seed bio-priming was conducted using the aforementioned liquid bacterial suspensions at 25 °C for 5 h, under dark conditions. After priming, seeds were rinsed thoroughly with distilled water, and dried on sterile filter paper at room temperature for 72 h [52].

### 3.4. Seed Quality and Vigor Testing

#### 3.4.1. Germination Test (GT)

The germination test was used to obtain the inoculated soybean seed quality under optimal conditions [53]. Samples consisted of 100 seeds per replicate, randomly selected. Seeds were sown in germination boxes 240 × 150 mm. Sterilized moist sand was used as the growing medium. Samples were placed into a germination chamber at 25 °C, with an illumination cycle of 12 h of light and 12 h of darkness for eight days. Energy of germination, defined as the percentage (%) by the number of seeds in a given sample, which germinate within a definite period, was determined five days after sowing by counting only the seedlings with well-developed essential structures [53]. The percentage of final germination (%), counting only seedlings with a healthy and well-developed root and shoot system, and percentage of abnormal seedlings (%), defined as seedlings which do not show potential for further development into satisfactory plants when grown in good quality soil and under favorable conditions of moisture, temperature and light, were determined eight days after sowing [53].

#### 3.4.2. Cold Test (CT)

The cold test was performed to determine the soybean seed’s ability to germinate under unfavorable conditions, such as low temperature. A total of fifty seeds per replicate were sown in a mixture of soil and sand in a 3:1 ratio [54]. Samples were exposed to the low temperature of 10 °C for seven days [54]. Subsequently, the samples were transferred to the germination chamber at 25 °C for six days [53]. Thereafter, seed germination and abnormal seedlings were determined in a single count [53].

#### 3.4.3. Accelerated Aging Test (AAT)

Accelerated aging test was performed in order to estimate the accurate vigor of inoculated soybean seeds. A total of 100 seeds of soybean per replicate were exposed to double stress, high temperature (42 °C) and high relative humidity (≈95%) for 72 h [54]. The germination test was conducted afterwards [53].

### 3.5. Determination of Seedlings Growth and Biomass Accumulation, and Seedling Vigor Index

Samples consisted of 25 randomly selected soybean seeds per replicate to measure shoot and root length. Samples were germinated in rolled filter paper for eight days in the germination chamber. Afterwards, 10 normal seedlings per replication were randomly selected for further assessment. In the cold test, samples were first exposed to a low temperature (10 °C) for seven days and then transferred into a germination chamber for six days. In the accelerated aging test, seeds were exposed to a high temperature and humidity for 72 h and then placed into a rolled filter paper for eight days in the germination chamber at 25 °C. The length of the shoot and roots were measured using a ruler on the day of germination. The fresh shoot and root weight of 10 seedlings were determined using analytical balance on the day of seed germination. For determination of dry weight, samples were oven dried at 80 °C for 24 h. The seedling vigor index was determined according to Abdul-Baki and Anderson [55], using the following formula:

SVI = (Shoot Length + Root Length) (cm) × Germination (%)

### 3.6. Statistical Analysis

Four treatments were arranged in a complete randomized design (CRD) for laboratory tests, with four replications. The obtained data were processed statistically, using analysis of variance (ANOVA), followed by mean separation according to Tukey’s HSD test (*p* ≤ 0.05). The relationship between germination and other parameters was determined by Pearson’s correlation analysis. Principal component analysis (PCA) was performed to determine the effect of seed bio-priming treatments on the examined parameters of soybean cultivars. The data were statistically processed by STATISTICA 10 programme (StatSoft Inc., Tulsa, OK, USA).

## 4. Conclusions

Overall, *Bacillus* and co-inoculation improved the majority of the investigated soybean parameters as compared to *Bradyrhizobium*. The greatest improvement was obtained for the shoot length, root length, and seedling vigor index in cold-treated seeds, followed by aged and normal seeds, as well as for shoot and root weight recorded in germination, cold and accelerated tests, respectively. Bio-priming treatments led to the highest increase in final germination in cold-treated seeds, whereas the effect was lower in normal and aged seeds. The present study represents the first experimental evidence of using newly-isolated, indigenous *B. megaterium* strains from soil, for seed bio-priming of soybean under different conditions of laboratory tests. This technique can be recommended for priming seeds prior to field planting as an environmentally friendly strategy to improve seed germination and initial seedling growth. Extensive field trials under different environmental conditions, using several seed lots of each soybean cultivar, will be necessary in order to establish the efficiency of these strains as potential bio-priming seed treatments for improving soybean productivity.

## Figures and Tables

**Table 1:** *Bacillus megaterium* strains isolated from soil at different locations in Serbia.

Strain	Source of Isolation	NCBI Accession Number
B8	Rhizosphere (pepper)	KU953928
B9	Rhizosphere (alfalfa)	KU953929
B12	Agricultural soil	KX444638
B14	Rhizosphere (sunflower)	KX444640
B15	Non-agricultural soil	KX444641
B16	Rhizosphere (maize)	KX444642
B17	Rhizosphere (wheat)	KX444643

**Table 2:** Plant growth-promoting properties of *Bacillus megaterium* strains.

Strain Code	IAA * (µg mL^−1^ ± SD) at 500 µg mL^−1^ L-Tryptophan	P Solubili- Zation	P Minerali-Zation	Growth on N- Free Medium	Sidero- Phores
B8	15.65 ± 0.13 a	+++	+++	+	++
B9	8.00 ± 0.12 e	nd	++	+	++
B12	12.30 ± 0.17 c	++	+++	+	++
B14	10.35 ± 0.11 d	+	+	+	nd
B15	13.70 ± 0.12 b	++	+++	+	+
B16	10.45 ± 0.15 d	nd	++	+	++
B17	12.48 ± 0.14 c	++	++	+	++

* Means (*n* = 3) with different lowercase letters in the same column are significantly different (*p* ≤ 0.05, Tukey’s HSD test); P solubilization/mineralization: (+) 1–4 mm of halo diameter, (++) 4–7 mm of halo diameter; (+++) 8–11 mm of halo diameter; (nd) not detected; Growth on N-free medium: (+) positive for the test; Siderophores: (+) 1–5 mm wide of orange zone, (++) 5–15 mm wide of orange zone, (nd) not detected.

**Table 3:** Analysis of variance for parameters of two soybean cultivars after applying *Bradyrhizobium japonicum* and *Bacillus megaterium* as a single inoculant and co-inoculant during seed bio-priming in different laboratory tests. (**a**) Germination Test; (**b**) Cold Test; (**c**) Accelerated Aging Test.

Factor	Traits							
	Germination Energy	Final Germination	Abnormal Seedlings	Shoot Length	Root Length	Shoot Dry Weight	Root Dry Weight	Seedling Vigor Index
	**Germination Test (a)**							
Cultivar (C)	ns	ns	ns	***	***	ns	***	***
Treatment (T)	***	***	***	***	***	***	***	***
C × T	**	**	**	***	***	*	***	***
	**Cold Test (b)**							
Cultivar (C)	-	ns	ns	***	***	***	*	***
Treatment (T)	-	***	***	***	***	***	***	***
C × T	-	ns	***	***	**	***	ns	***
	**Accelerated Aging Test (c)**							
Cultivar (C)	ns	**	***	***	ns	ns	*	***
Treatment (T)	***	***	***	***	***	*	*	***
C × T	***	***	***	***	***	ns	***	***

* *p* ≤ 0.05, ** *p* ≤ 0.01, *** *p* ≤ 0.001, ns—not significant.

**Table 4:** Effect of seed bio-priming treatments on soybean parameters in different laboratory tests. (**a**) Germination Test; (**b**) Cold Test; (**c**) Accelerated Aging Test.

Treatment	Traits							
	Germination Energy (%)	Final Germination (%)	Abnormal Seedlings (%)	Shoot Length (mm)	Root Length (mm)	Shoot Dry Weight (g)	Root Dry Weight (g)	Seedling Vigor Index
**Germination Test (a)**								
Control	76.38 b	87.25 b	8.88 a	118.42 b	144.63 b	0.825 b	0.121 b	2297.07 c
*Br. japonicum* (BJ)	79.75 a	88.63 ab	8.25 ab	128.18 a	147.07 b	0.963 a	0.178 a	2439.13 b
*B. megaterium* (BM)	79.13 a	90.75 a	6.88 bc	127.51 a	157.50 a	0.981 a	0.193 a	2584.51 a
BJ + BM	78.63 a	90.50 a	6.13 c	127.08 a	154.57 a	0.837 b	0.206 a	2548.03 a
**Cold Test (b)**								
Control	-	77.00 b	16.75 a	59.63 b	72.63 b	1.057 b	0.055 b	1018.49 b
*Br. japonicum* (BJ)	-	83.00 a	12.00 c	78.38 a	101.50 a	1.123 a	0.078 a	1494.62 a
*B. megaterium* (BM)	-	80.38 a	14.63 b	78.69 a	104.38 a	1.142 a	0.082 a	1470.99 a
BJ + BM	-	82.13 a	14.00 b	80.56 a	103.63 a	1.155 a	0.074 a	1512.70 a
**Accelerated Aging Test (c)**								
Control	73.00 b	79.63 b	11.63 b	118.07 d	103.38 c	0.959 ab	0.157 b	1768.39 c
*Br. japonicum* (BJ)	69.25 c	77.00 c	16.63 a	127.44 c	130.69 b	0.917 ab	0.177 ab	1989.74 b
*B. megaterium* (BM)	75.00 ab	82.88 a	12.50 b	134.12 b	148.63 a	0.903 b	0.184 a	2342.21 a
BJ + BM	76.13 a	81.13 ab	15.50 a	141.50 a	136.19 b	0.969 a	0.167 ab	2256.65 a

Data are represented as mean (*n* = 4); Differences between treatments were analyzed using the Tukey’s HSD test (*p* ≤ 0.05). Means within each trait followed by the same letters are not significantly different.

**Table 5:** Pearson correlations between germination and other parameters of soybean. (**a**) Germination Test; (**b**) Cold Test; (**c**) Accelerated Aging Test.

Traits	Abnormal Seedlings	Shoot Length	Root Length	Shoot Dry Weight	Root Dry Weight	Seedling Vigor Index
	**Germination Test (a)**					
Germination energy	−0.404 *	0.315 ns	0.046 ns	0.515 **	0.301 ns	0.335 ns
Final germination	−0.685 ***	0.243 ns	0.189 ns	0.380 *	0.416 *	0.606 ***
	**Cold Test (b)**					
Final germination	−0.619 ***	0.603 ***	0.604 ***	0.323 ns	0.493 **	0.781 ***
	**Accelerated Aging Test (c)**					
Germination energy	−0.531 **	0.441 *	0.202 ns	0.187 ns	0.094 ns	0.458 **
Final germination	−0.631 ***	0.519 **	0.287 ns	0.001 ns	0.289 ns	0.599 ***

* *p* ≤ 0.05, ** *p* ≤ 0.01, *** *p* ≤ 0.001, ns—not significant.

## Data Availability

Dataset supporting reported results are available at ECOBREED Zenodo community: 10.5281/zenodo.5521308.

## Supplementary Materials

The following supporting information can be downloaded at: https://www.mdpi.com/article/10.3390/plants11151927/s1, Table S1: Analysis of variance for parameters of two soybean cultivars after applying *Bradyrhizobium japonicum* and *Bacillus megaterium* as a single inoculant and co-inoculant during seed bio-priming in different laboratory tests. Table S2: Effect of seed bio-priming treatments on soybean parameters in germination test. Table S3: Effect of seed bio-priming treatments on soybean parameters in cold test. Table S4: Effect of seed bio-priming treatments on soybean parameters in accelerated aging test.
