# Supplementary material for: Bio-Priming of Soybean with Bradyrhizobium japonicum and Bacillus megaterium: Strategy to Improve Seed Germination and the Initial Seedling Growth
Source: Plants (Basel). 2022 Jul 25;11(15):1927. doi: 10.3390/plants11151927 (PMC9332681; doi:10.3390/plants11151927)
Supplement: Supplementary file 1 [file plants-11-01927-s001.zip › plants-1757348-supplementary.pdf]

Supplementary Table S1. Analysis of variance for parameters of two soybean cultivars after applying *Bradyrhizobium japonicum* and *Bacillus megaterium* as a single inoculant and co-inoculant during seed bio-priming in different laboratory tests.

Factor	Traits																							
	Germination energy			Final Germination			Abnormal seedlings			Shoot length			Root length			Shoot dry weight			Root dry weight			Seedling vigor index		
**Germination Test (a)**																								
	df	*F*	*p*	*df*	F	p	*df*	*F*	*p*	*df*	*F*	*p*	*df*	*F*	*p*	*df*	*F*	*p*	*df*	*F*	*p*	*df*	*F*	*p*
Cultivar (C)	1	0.2	0.7012	1	0.5	0.4686	1	3.3	0.0805	1	23.4	0.0001	1	188.5	0.0000	1	1.7	0.2075	1	36.3	0.0000	1	44.9	0.0000
Treatment (T)	3	9.3	0.0003	3	7.8	0.0009	3	11.1	0.0001	3	15.5	0.0000	3	15.2	0.0000	3	14.3	0.0000	3	17.4	0.0000	3	32.8	0.0000
C × T	3	7.3	0.0012	3	5.2	0.0069	3	6.2	0.0029	3	8.9	0.0004	3	18.1	0.0000	3	3.1	0.0446	3	13.2	0.0000	3	15.2	0.0000
**Cold Test (b)**																								
Cultivar (C)	-	-	-	1	1.1	0.3053	1	0.6	0.4600	1	22.8	0.0001	1	234.3	0.0000	1	30.1	0.0000	1	5.9	0.0222	1	26.1	0.0000
Treatment (T)	-	-	-	3	10.1	0.0002	3	22.1	0.0000	3	95.3	0.0000	3	313.2	0.0000	3	14.2	0.0000	3	16.7	0.0000	3	221.1	0.0000
C × T	-	-	-	3	1.8	0.1689	3	31.6	0.0000	3	7.9	0.0008	3	5.1	0.0075	3	14.2	0.0000	3	2.1	0.1325	3	9.6	0.0002
**Accelerated Aging Test (c)**																								
Cultivar (C)	1	0.2	0.6443	1	8.4	0.0079	1	45.3	0.0000	1	73.3	0.0000	1	1.5	0.2294	1	3.2	0.0871	1	5.8	0.0238	1	49.9	0.0000
Treatment (T)	3	20.4	0.0000	3	13.9	0.0000	3	41.1	0.0000	3	51.7	0.0000	3	84.5	0.0000	3	4.7	0.0106	3	3.5	0.0313	3	111.4	0.0000
C × T	3	23.7	0.0000	3	12.5	0.0000	3	36.0	0.0000	3	46.2	0.0000	3	130.0	0.0000	3	1.6	0.2221	3	12.8	0.0000	3	136.9	0.0000

Supplementary Table S2. Effect of seed bio-priming treatments on soybean parameters in germination test.

Treatment	Cultivar	
	**Atlas**	**Teona**
	Germination Energy (%)	
Control	74.75 ± 0.25 bB	78.00 ± 0.71 aA
B. japonicum (BJ)	80.25 ± 1.03 aA	79.25 ± 0.95 aA
B. megaterium (BM)	80.50 ± 0.29 aA	77.75 ± 0.48 aAB
BJ + BM	78.00 ± 0.82 aA	79.25 ± 0.48 aA
Average	78.38 A	78.56 A
	Final Germination (%)	
Control	85.25 ± 1.31 bB	89.25 ± 0.95 abA
B. japonicum (BJ)	89.25 ± 0.63 abA	88.00 ± 0.41 bAB
B. megaterium (BM)	91.75 ± 0.63 aA	89.75 ± 0.25 abA
BJ + BM	90.00 ± 1.22 aA	91.00 ± 0.71 aA
Average	89.06 A	89.50 A
	Abnormal Seedlings (%)	
Control	9.75 ± 0.63 aA	8.00 ± 0.71 aAB
B. japonicum (BJ)	8.00 ± 0.41 abAB	8.50 ± 0.29 aAB
B. megaterium (BM)	6.25 ± 0.63 bcBC	7.50 ± 0.65 aAB
BJ + BM	4.75 ± cC	7.50 ± 0.29 aAB
Average	7.19 A	7.88 A
	Shoot Length (mm)	
Control	118.83 ± 0.83 cD	118.00 ± 1.67 bD
B. japonicum (BJ)	132.18 ± 1.39 abA	124.18 ± 1.05 abBCD
B. megaterium (BM)	127.18 ± 1.53 bABC	127.83 ± 2.74 aAB
BJ + BM	134.33 ± 1.16 aA	119.83 ± 2.05 abCD
Average	128.13 A	122.46 B
	Root Length (mm)	
Control	132.88 ± 2.02 bC	156.38 ± 0.43 bB
B. japonicum (BJ)	137.88 ± 3.18 bC	156.25 ± 2.57 bB
B. megaterium (BM)	138.50 ± 2.05 bC	176.50 ± 0.35 aA
BJ + BM	151.63 ± 2.55 aB	157.50 ± 2.68 bB
Average	140.22 B	161.66 A
	Shoot Dry Weight (g)	
Control	0.765 ± 0.035 cB	0.884 ± 0.047 abAB
B. japonicum (BJ)	0.957 ± 0.026 aA	0.968 ± 0.052aA
B. megaterium (BM)	0.957 ± 0.009 aA	1.004 ± 0.015 aA
BJ + BM	0.869 ± 0.017 bAB	0.804 ± 0.017 bB
Average	0.887 A	0.915 A
	Root Dry Weight (g)	
Control	0.114 ± 0.012 cC	0.128 ± 0.016 bBC
B. japonicum (BJ)	0.186 ± 0.005 aB	0.169 ± 0.007 bBC
B. megaterium (BM)	0.140 ± 0.010 bcBC	0.246 ± 0.010 aA
BJ + BM	0.150 ± 0.003 bBC	0.262 ± 0.024 aA
Average	0.147 B	0.201 A
	Seedling Vigor Index	

Control	2145.13 ± 24.45 cD	2449.01 ± 35.52 bBC
B. japonicum (BJ)	2410.82 ± 53.79 bC	2467.43 ± 22.84 bBC
B. megaterium (BM)	2437.70 ± 27.28 abBC	2731.32 ± 24.08 aA
BJ + BM	2572.80 ± 20.99 aB	2523.26 ± 33.15 bBC
Average	2391.61 B	2542.76 A

Data are represented as mean ± standard error od the mean (n=4); Differences between treatments were analysed using the Tukey's HSD test ($p \leq 0.05$). Means within each trait followed by the same lowercase/uppercase letters are not significantly different.

Supplementary Table S3. Effect of seed bio-priming treatments on soybean parameters in cold test.

Treatment	Cultivar	
	**Atlas**	**Teona**
	Final Germination (%)	
Control	76.00 ± 2.16 bC	78.00 ± 1.41 bBC
B. japonicum (BJ)	81.25 ± 0.48 aABC	84.75 ± 0.48 abA
B. megaterium (BM)	80.75 ± 0.48 abABC	80.00 ± 0.82 abABC
BJ + BM	82.75 ± 0.95 aAB	81.50 ± 1.50 abABC
Average	80.19 A	81.06 A
	Abnormal Seedlings (%)	
Control	18.75 ± 0.48 aA	14.75 ± 0.48 abBC
B. japonicum (BJ)	13.25 ± 0.48 cCD	10.75 ± 0.48 cD
B. megaterium (BM)	15.25 ± 0.25 bBC	14.00 ± 0.82 bC
BJ + BM	10.75 ± 0.48 dD	17.25 ± 0.95 aAB
Average	14.50 A	14.19 A
	Shoot Length (mm)	
Control	62.13 ± 1.01 cD	57.13 ± 1.23 bD
B. japonicum (BJ)	77.25 ± 0.95 bBC	79.50 ± 1.74 aABC
B. megaterium (BM)	84.50 ± 1.08 aA	72.88 ± 2.59 aC
BJ + BM	83.00 ± 0.94 aAB	78.13 ± 1.01 aABC
Average	76.72 A	71.91 B
	Root Length (mm)	
Control	67.13 ± 0.43 cE	78.13 ± 1.65 bD
B. japonicum (BJ)	92.88 ± 1.48 bC	110.13 ± 0.31 aA
B. megaterium (BM)	96.50 ± 1.97 abBC	112.25 ± 1.25 aA
BJ + BM	99.13 ± 0.31 aB	108.13 ± 1.13 aA
Average	88.91 B	102.16 A
	Shoot Dry Weight (g)	
Control	1.053 ± 0.035 aCD	1.061 ± 0.008 cD
B. japonicum (BJ)	1.135 ± 0.017 aBC	1.110 ± 0.016 bCD
B. megaterium (BM)	1.091 ± 0.004 aCD	1.192 ± 0.013 aAB
BJ + BM	1.070 ± 0.010 aCD	1.239 ± 0.006 aA
Average	1.088 B	1.151 A
	Root Dry Weight (g)	
Control	0.049 ± 0.004 bC	0.061 ± 0.003 bBC
B. japonicum (BJ)	0.077 ± 0.008 aAB	0.078 ± 0.001 aAB
B. megaterium (BM)	0.082 ± 0.002 aA	0.081 ± 0.003 aA
BJ + BM	0.066 ± 0.003 abABC	0.081 ± 0.002 aA
Average	0.068 B	0.075 A
	Seedling Vigor Index	
Control	982.67 ± 32.52 cD	1054.30 ± 11.58 cD
B. japonicum (BJ)	1382.42 ± 17.65 bC	1606.81 ± 10.27 aA
B. megaterium (BM)	1461.62 ± 25.59 abBC	1480.35 ± 6.25 bBC
BJ + BM	1506.80 ± 10.91 aAB	1518.60 ± 40.84 abAB
Average	1333.38 B	1415.02 A

DData are represented as mean ± standard error od the mean (n=4); Differences between treatments were analysed using the Tukey's HSD test ($p \leq 0.05$). Means within each trait followed by the same lowercase/uppercase letters are not significantly different.

Supplementary Table S4. Effect of seed bio-priming treatments on soybean parameters in accelerated aging test.

Treatment	Cultivar	
	**Atlas**	**Teona**
	Germination Energy (%)	
Control	71.00 ± 0.71 cBC	75.00 ± 1.87 aB
B. japonicum (BJ)	67.25 ± 0.95 dC	71.25 ± 0.95 aBC
B. megaterium (BM)	74.75 ± 0.25 bB	75.25 ± 0.85 aB
BJ + BM	81.00 ± 0.41 aA	71.25 ± 0.63 aBC
Average	73.50 A	73.19 A
	Final Germination (%)	
Control	78.25 ± 1.18 bBC	81.00 ± 0.41 aAB
B. japonicum (BJ)	78.25 ± 1.25 bBC	75.75 ± 0.48 bC
B. megaterium (BM)	82.75 ± 1.25 abA	83.00 ± 1.08 aA
BJ + BM	85.25 ± 0.95 aA	77.00 ± 0.41 bBC
Average	81.13 A	79.19 B
	Abnormal Seedlings (%)	
Control	13.00 ± 0.82 bCD	10.25 ± 0.25 dE
B. japonicum (BJ)	16.25 ± 0.48 aB	17.00 ± 0.82 bAB
B. megaterium (BM)	10.25 ± 0.48 cE	14.75 ± 0.48 cBC
BJ + BM	11.75 ± 0.25 bcDE	19.25 ± 0.25 aA
Average	12.81 B	15.31 A
	Shoot Length (mm)	
Control	110.13 ± 2.18 cE	126.00 ± 1.62 abCD
B. japonicum (BJ)	136.13 ± 1.50 bB	118.75 ± 2.05 bDE
B. megaterium (BM)	147.10 ± 2.58 aA	121.13 ± 2.87 bD
BJ + BM	151.50 ± 0.20 aA	131.50 ± 1.41 aBC
Average	136.21 A	124.34 B
	Root Length (mm)	
Control	72.75 ± 2.14 cE	134.00 ± 1.67 aBCD
B. japonicum (BJ)	141.13 ± 2.47 bB	120.25 ± 3.24 bD
B. megaterium (BM)	174.00 ± 2.16 aA	123.25 ± 5.10 abCD
BJ + BM	136.13 ± 3.65 bBC	136.25 ± 1.01 aBC
Average	131.00 A	128.44 A
	Shoot Dry Weight (g)	
Control	0.951 ± 0.047 aA	0.967 ± 0.023 aA
B. japonicum (BJ)	0.878 ± 0.012 aA	0.956 ± 0.002 aA
B. megaterium (BM)	0.891 ± 0.012 aA	0.914 ± 0.008 aA
BJ + BM	0.974 ± 0.005 aA	0.963 ± 0.019 aA
Average	0.924 A	0.950 A
	Root Dry Weight (g)	
Control	0.133 ± 0.006 cC	0.180 ± 0.017 aAB
B. japonicum (BJ)	0.192 ± 0.003 abAB	0.161 ± 0.004 aBC
B. megaterium (BM)	0.214 ± 0.005 aA	0.153 ± 0.008 aBC
BJ + BM	0.175 ± 0.008 bAB	0.158 ± 0.012 aBC

Average	0.179 A	0.163 B
	Seedling Vigor Index	
Control	1430.56 ± 34.14 dE	2106.21 ± 25.09 aC
B. japonicum (BJ)	2169.18 ± 28.69 cC	1810.30 ± 8.49 bD
B. megaterium (BM)	2655.63 ± 14.24 aA	2028.98 ± 73.62 aC
BJ + BM	2451.49 ± 30.57 bB	2061.81 ± 23.72 aC
Average	2176.72 A	2001.83 B

Data are represented as mean ± standard error od the mean (n=4); Differences between treatments were analysed using the Tukey's HSD test ($p \leq 0.05$). Means within each trait followed by the same lowercase/uppercase letters are not significantly different.
